# Critical Dynamics in the Association Cortex Predict Higher Intelligence in Typically Developing Children

**DOI:** 10.1523/JNEUROSCI.1414-25.2026

**Published:** 2026-02-02

**Authors:** Gianina Cristian, Cece C. Kooper, Arthur-Ervin Avramiea, Jennifer R. Ramautar, Jordache Ramjith, Shilpa Anand, Marsh Königs, Gert Jan van der Wilt, Hilgo Bruining, Klaus Linkenkaer-Hansen

**Affiliations:** ^1^Donders Institute for Brain, Cognition and Behaviour, Radboud University Medical Center, Nijmegen 6500 HD, The Netherlands; ^2^IQ Health, Radboud University Medical Center, Nijmegen 6525 GA, The Netherlands; ^3^N=You Neurodevelopmental Precision Center, Amsterdam Neuroscience, Amsterdam Reproduction and Development, Amsterdam UMC, Amsterdam 1105 AZ, The Netherlands; ^4^Child and Adolescent Psychiatry and Psychosocial Care, Emma Children’s Hospital, Amsterdam UMC, VU University Amsterdam, Amsterdam 1105 AZ, The Netherlands; ^5^Levvel, Academic Center for Child and Adolescent Psychiatry, Amsterdam 1007 MB, The Netherlands; ^6^Amsterdam Reproduction and Development Research Institute, Amsterdam 1105 AZ, The Netherlands; ^7^Department of Pediatrics, Follow-Me program & Emma Neuroscience Group, Emma Children’s Hospital, Amsterdam UMC, University of Amsterdam, Amsterdam 1105 AZ, The Netherlands; ^8^Department of Integrative Neurophysiology, Center for Neurogenomics and Cognitive Research (CNCR), Amsterdam Neuroscience, VU University Amsterdam, Amsterdam 1081 HV, The Netherlands; ^9^Emma Center for Personalized Medicine, Amsterdam UMC, Amsterdam 1105 AZ, The Netherlands; ^10^Department of Medical Microbiology, Radboud University Nijmegen Medical Center, Nijmegen 6525 GA, The Netherlands

**Keywords:** cognition, criticality, electroencephalography, excitation–inhibition balance, intelligence, neurodevelopment

## Abstract

Neuronal network models have indicated that the so-called critical dynamics facilitate efficient information processing, while criticality disruptions were linked to neuropathology through excitation/inhibition (E/I) imbalances. However, there is limited empirical evidence for a relationship between critical brain dynamics and cognition in healthy children and adolescents. Here, we investigate how these dynamics relate to intelligence in a developing cohort. We recorded eyes-open resting EEG in 128 children (6–19 years, 72 female) and quantified near-critical dynamics in the alpha-band using functional excitation/inhibition ratio (*f*E/I) and in nonoscillatory activity using the 1/*f* aperiodic exponent of the power spectrum. We devised models relating intelligence to *f*E/I and 1/*f* exponent across seven Yeo7 functional brain networks ranked from lower-order sensorimotor to higher-order association networks. We observed significant correlations between *f*E/I and 1/*f* exponent and IQ in association cortices, in contrast to sensorimotor cortices. Children in the high-IQ group had *f*E/I ratios closer to the theoretical critical value of 1 in association cortices compared with the low-IQ group. The association–sensorimotor axis rank moderated the associations between 1/*f* exponent and IQ, these associations decreasing on a gradient across the hierarchy of the Yeo7 networks. Age and rank moderated the *f*E/I–IQ association, with the association–sensorimotor effect size gradient most visible in adolescents. Together, the results suggest that individual variation in criticality-sensitive biomarkers in association networks may be linked to IQ differences in an age-dependent manner, consistent with the hypothesis that developmental modulation of critical dynamics across the cortical hierarchy may support more efficient cognitive processing.

## Significance Statement

The healthy brain is posited to operate near a critical transition between a subcritical state, characterized by excessive neural inhibition, and a super-critical state, marked by excessive neural excitability. Preclinical and computational modeling studies have shown that this critical state is conducive to optimal information processing. The present study provides insight, using electroencephalographic (EEG) brain recordings, into how brain criticality is linked to intelligence during development. The study offers important empirical evidence in agreement with computational studies linking brain criticality to optimal functioning and may help to better understand the role of criticality in brain disorders.

## Introduction

According to the theory of critical brain dynamics, the healthy brain operates near a critical transition between a subcritical state, characterized by excessive neural inhibition, and a super-critical state, marked by excessive excitability (Beggs, 2008; Chialvo, 2010; Shew and Plenz, 2013; Cocchi et al., 2017; Zimmern, 2020; O’Byrne and Jerbi, 2022; Hengen and Shew, 2025). Preclinical and computational studies have suggested that proximity to criticality enhances information processing, maximizing computational efficiency and adaptive responsiveness to stimuli (Kinouchi and Copelli, 2006; Shew et al., 2009; Shew et al., 2015; Avramiea et al., 2020; Avramiea et al., 2022). Conversely, deviations from criticality have been implicated in neuropsychiatric disorders, particularly those involving excitation/inhibition (E/I) imbalances (Rubenstein and Merzenich, 2003; Gao and Penzes, 2015; Zhou and Yu, 2018; Ma et al., 2019; Zeraati et al., 2023). Despite increasing interest in criticality as a mechanism underlying cognition and pathology, the relationship between critical brain dynamics and information processing, here operationalized as intelligence quotient (IQ), remains understudied, particularly in children.

A key mechanism governing criticality is the balance between excitatory (E) and inhibitory (I) signaling, with the E/I ratio modulating neural systems near criticality (Beggs and Timme, 2012). Computational modeling shows that at specific E/I ratios, networks exhibit scale-free temporal structure, including long-range temporal correlations with power-law decay, consistent with dynamics near-critical regimes (Linkenkaer-Hansen et al., 2001; Linkenkaer-Hansen et al., 2007; Hardstone et al., 2012; Poil et al., 2012; Zhigalov et al., 2015). However, long-range temporal correlation strength alone does not distinguish sub- from super-critical states. A new functional E/I (*f*E/I) metric quantifies the covariance between long-range temporal correlation strength and oscillatory amplitude. *f*E/I values near 1 are interpreted as consistent with proximity to a critical regime, with deviations indicating super- or subcriticality (Bruining et al., 2020).

While *f*E/I captures dynamic fluctuations in oscillatory activity, the 1/*f* spectral exponent provides a complementary, potentially E/I-sensitive measure of aperiodic background activity. Neuronal populations generate oscillations in the EEG signal superimposed on a nonoscillatory 1/*f*^β^ background with power-law exponent β (Freeman and Zhai, 2009; He et al., 2010; Donoghue et al., 2020). Theory links the 1/*f* exponent to self-organized criticality (Bak et al., 1987; Beggs and Plenz, 2003; Priesemann, 2014). The exponent has been related to cortical excitability and E/I balance in preclinical studies (Gao et al., 2017) and implicated in neuropsychiatric disorders (Ostlund et al., 2021; Donoghue, 2025).

Importantly, regional and developmental variation in criticality-sensitive measures is observed in healthy populations, underscoring the need for normative regional and developmental groundwork, alongside disorder-focused work (Linkenkaer-Hansen et al., 2005; Montez et al., 2009; Liu et al., 2014; Alamian et al., 2022; Toker et al., 2022; Sihn et al., 2023). Regionally, critical dynamics may be organized along the sensorimotor–association axis, consistent with hierarchy models spanning unimodal sensorimotor systems and transmodal association networks (frontoparietal and default mode) implicated in more integrative cognition (Sydnor et al., 2021; Sydnor et al., 2023). Developmentally, criticality-sensitive measures may show age-related change (Smit et al., 2011; Hu et al., 2023; Sydnor et al., 2025) in a manner broadly consistent with sequential cortical maturation, described as earlier maturation of primary sensorimotor, followed by association networks (Dong et al., 2021; Larsen et al., 2022; Zhang et al., 2024). These spatial and developmental gradients motivate a network-based, developmental framework for relating criticality to intelligence.

Although critical dynamics have been linked to efficient information processing, evidence relating them to intelligence remains sparse especially in children and has largely relied on fMRI (Ezaki et al., 2020; Xu et al., 2022), whereas critical signatures such as long-range temporal correlations are inherently temporal and most directly expressed in electrophysiological signals captured with electroencephalography (EEG).

We hypothesize that resting-state critical dynamics associate with IQ in children and are modulated by development and network organization along the sensorimotor–association axis; in case of *f*E/I, proximity to the theoretical critical regime would generally be associated with higher IQ scores. To test this, we examine associations between EEG-derived criticality-sensitive biomarkers, *f*E/I and the 1/*f* exponent, and intelligence in children. By characterizing normative childhood patterns of critical brain dynamics, we address a developmental gap to inform future neuropsychiatric disorder research.

## Materials and Methods

### Study population and procedure

A community children sample was recruited from Amsterdam University Medical Center (AMC) networks and primary and secondary schools, sports, and social organizations in the Netherlands between July 2021 and May 2023. More information is available in Königs et al. (2023). A visit to Emma Children’s Hospital or a mobile laboratory “Emma Brain Bus” was planned after verbal consent. Written consent was collected before testing. We obtained ethical approval from the Medical Ethical Committee of the Amsterdam UMC (NL76915.018.21) and registered the trial with the International Clinical Trials Registry Platform (NL9574). To uniformly represent various age groups, we targeted 20 subjects in each 2-year category in the range 6–19 years, resulting in 129 subjects. The inclusion criteria were age 6–19 years (mean, 11.6 *±* 3.5), native Dutch language, and Netherlands residency. Exclusion criteria were an inability to understand test instructions or sensory-motor impairments preventing neurocognitive testing. We excluded *n* = 1 subject due to a bad EEG signal, totaling 128 subjects (72 female).

### Intelligence

Intelligence (IQ) was assessed (Königs et al., 2023) using a short form of the Wechsler Intelligence Scale (Wechsler, 2012) comprising Vocabulary, Similarities, Block Design, and Matrix Reasoning tests. The full-scale IQ could be estimated using the short form with a high validity and reliability (rs ≥ 0.82 and *r* ≥ 0.92, respectively; Sattler, 2001). The Wechsler Intelligence Scale V (Wechsler, 2012, 2014) was used for children aged 6–16 and Scale IV (Wechsler, 2008) for children aged 17–19.

### EEG recording and preprocessing

We recorded EEG for 5 min during eye-open rest using the high-density 128-channel HydroCel Geodesic with the NetAmps400 amplifier (Magstim-Electrical Geodesics). The EEGs were preprocessed using a semiautomated EEGLAB preprocessing pipeline (S.A.) in EEGLAB (v2022.0v; MATLAB R2021b; Fischler and Bolles, 1981; Bell and Sejnowski, 1995; Delorme and Makeig, 2004; Miyakoshi, 2023). For details, see Supplemental Material. Residual artifacts of noisy channels and segments were flagged in a machine learning step (Diachenko et al., 2022; Weiler et al., 2023) and removed by J.R.R. who visually inspected the signals. After preprocessing, the average signal duration was 194 ± 54 s; range, 64–289 s) for the cohort. Subsequently, we rereferenced the data to the average reference.

### Functional networks

**Figure 1.**
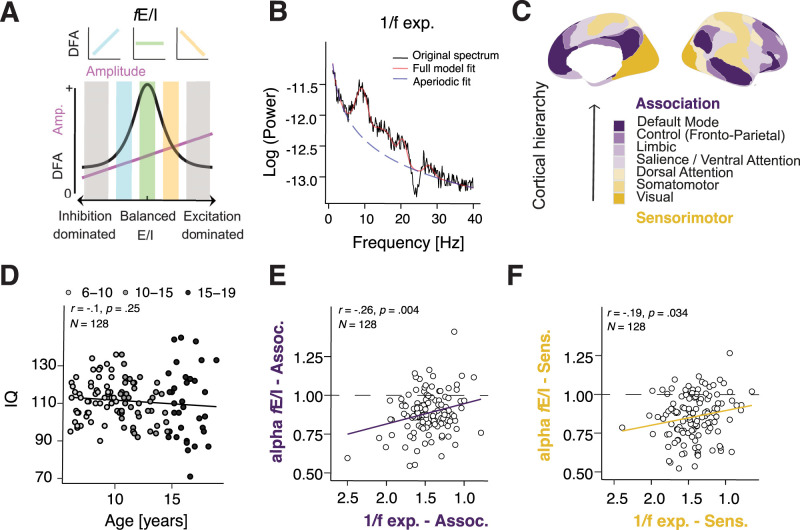
Quantifying near-critical brain dynamics from EEG in children (6–19 years) using the functional excitation/inhibition ratio (*f*E/I) and the 1/*f* aperiodic exponent. ***A***, We characterized the source EEG signals using *f*E/I and 1/*f* exponent. Computational proximity to criticality reflects a balanced E/I, with *f*E/I = 1, and maximizes long-range temporal correlations as determined with detrended fluctuation analysis (DFA). If *f*E/I < 1, the network is subcritical, or inhibition dominated. If *f*E/I > 1, the network is super-critical or excitation dominated*.* Reproduced from Bruining et al. (2020) with permission*.* ***B***, We computed the β exponent of the 1/*f*^β^ function, quantifying aperiodic activity beneath oscillatory peaks; with smaller exponent values previously linked to a higher E/I. ***C***, Eyes-open rest EEG was recorded for 5 min, source modeled, and grouped into seven resting-state functional networks of the Yeo7 atlas. ***D***, IQ showed no association with age. ***E***, *f*E/I and 1/*f* exponents showed modest but significant correlations in association and ***F***, sensorimotor regions.

The sensorimotor–association axis ranges from the unimodal, visual and somatomotor networks, supporting basic sensorimotor functions, to higher-order, association networks such as the frontoparietal (Control) and Default Mode networks, involved in complex cognition (Keller et al., 2023). To relate the scalp EEG to anatomical sources, the source time series were grouped into the 14 cortical patches (7 in the left, and 7 in the right hemisphere) based on the Yeo7 atlas for the primary analyses. For broad regional contrasts, we grouped these networks into association and sensorimotor networks according to their position in the sensorimotor–association cortical hierarchy (Sydnor et al., 2021; Sydnor et al., 2023; Zhang et al., 2024; Fig. 1*C*). To test the sensitivity of the results to the source reconstruction method, we replicated the analyses in sensor space as follows: we averaged the computed biomarker values per sensor into seven broadly analogous local regions ranked 1 to 7 and two broadly analogous sensorimotor and association regions (Table S2*B*). Furthermore, to test the sensitivity of the results to the parcellation method, we grouped the source time series into 68 Desikan–Killiany (Desikan et al., 2006) cortical patches numbered 1–68 according to their rank on the sensorimotor–association cortical hierarchy as in Zhang et al. (2024) (Table S2*A*).

### EEG analysis

#### Biomarker algorithms

Computational neuronal network models have shown that changes in the balance between excitatory and inhibitory signaling regulate network criticality, differentially impacting the spectral power, long-range temporal correlations, and functional excitation–inhibition ratio (*f*E/I). Here, we applied the *f*E/I algorithm optimized for narrow-band application on two log-spaced low and high alpha bands (8.3–10.5 and 10.5–13.4 Hz, respectively; Diachenko et al., 2024). Log-spaced frequency binning optimizes the trade-off between high spectral resolution, desirable for DFA computation, and data redundancy due to filter overlap. When computing *f*E/I on individual alpha frequencies, to allow analogy with the low- and high- alpha frequency bins, each individualized alpha band was split into two equal half-bands and biomarkers computed separately for each.

Excitation–inhibition ratio (E/I) was conceptualized as the control parameter of biological systems which operate at criticality (Beggs, 2008). According to computational modeling, the balance of excitatory (E) and inhibitory (I) signaling in the network producing the neuronal oscillation influences the strength of long-range temporal correlations (LRTCs) of oscillations, informing the distance to criticality (Hardstone et al., 2012; Poil et al., 2012). LRTCs are complex fluctuations in amplitude exhibited by neuronal oscillations, which are characterized by a power-law decay of autocorrelations. Briefly stated, simulations indicate that when networks are tuned from an inhibition-dominated, subcritical state to an excitation-dominated, super-critical state, the spectral power goes monotonously up, without a clear hallmark of the critical point. In contrast, the power-law exponent of the detrended fluctuation analysis (DFA) quantifies long-range temporal correlations and approaches 1.0 as networks become critical and drops toward 0.5, when networks become either sub- or super-critical. Therefore, the DFA does not distinguish well between sub- and super-critical networks. The *f*E/I algorithm solves this problem by analyzing the covariance of spectral power and long-range temporal correlations and returns a value of < 1.0 for subcritical networks, 1.0 for critical networks, and > 1.0 for super-critical networks (Bruining et al., 2020).

#### Functional network E/I ratio (*f*E/I)

The algorithm for estimating E/I ratio was derived from the Critical Oscillations (CROS) model of ongoing neuronal activity (Poil et al., 2012). The CROS model showed a strong association between the ratio of excitatory/inhibitory connectivity, LRTC, and oscillation amplitude. From these observations, we estimated a functional form of E/I ratio (*fE*/*I*) based on the correlation between the amplitude and LRTC in the signal. To test the relationship between the amplitude and LRTC of the amplitude envelope of an oscillatory signal, a measure of LRTC on short time-scales was needed, which is unbiased by the signal amplitude. To this end, an amplitude-normalized fluctuation function, *nF*(*t*), was calculated in the following way: (1) the signal was bandpass filtered, (2) the amplitude envelope *A* extracted (3) the signal profile, *S* was then calculated as the cumulative sum of the demeaned amplitude envelope:

$$S(t)=\overset{t}{\underset{k=1}{\sum }}\;\left(A(k)-\left\langle A\right\rangle \right),$$

and split into windows of a certain size (the default being 5 s) in exactly the same way as during the DFA calculation (Supplemental Material). (4) As an additional step, each of these signal-profile windows is divided by the mean of the amplitude envelope for that window calculated during step (2). (5) These amplitude-normalized windows are then detrended, and subsequently, (6) the normalized fluctuation function is calculated for each window as the standard deviation of the amplitude-normalized signal profile. (7) To calculate the functional excitation/inhibition ratio, *f*E/I, the Pearson’s correlation between the amplitude and the normalized fluctuation function for the set of windows *W* is performed. *f*E/I is then defined as follows:

$$fE/I=1-r_{\mathrm{Wamp},WnF(t)},$$

if *f*E/I = 1, the network is thought to be operating in a critical state. If *f*E/I < 1, the network is subcritical, or inhibition dominated. If *f*E/I > 1, the network is super-critical or excitation dominated. Since networks without LRTC (DFA < 0.6) will not exhibit covariation of amplitude and fluctuation function, we used a DFA > 0.6 criterion for computing *f*E/I. Thus, *f*E/I computation is limited to networks exhibiting near-critical dynamics, as indicated by significant long-range temporal correlations, i.e., DFA exponent > 0.6 (Bruining et al., 2020; Diachenko et al., 2024).

#### DFA parametrization

The DFA > 0.6 criterion follows the validated procedure described in Diachenko et al. (2024). In simulations, networks with unbalanced E/I ratio exhibit a DFA <0.6, and amplitude-envelope segments with DFA ≤ 0.6 no longer exhibit the covariance structure between amplitude and fluctuations (Fig. 1*A*). Random signals have a DFA exponent of 0.5, while white-noise signals have a DFA exponent <0.6. The added technical limitation of finite signal duration, however, leads to variation in DFA exponents, making an exponent <0.6 a more reliable indicator of random fluctuations than <0.5 in empirical data. We thus treated DFA ≤ 0.6 as indicating insufficient long-range temporal structure for reliable *f*E/I computation, without interpreting these values physiologically, but rather as a methodological limitation.

#### *f*E/I parametrization

In this paper, *f*E/I was calculated for windows of 5 s with 80% overlap. We based this parametrization on the sensitivity analysis by Diachenko et al. (2024). *f*E/I accuracy, i.e., the extent to which the *f*E/I indexes critical dynamics, was measured as the decrease in the mean absolute rank change between *f*E/I and the avalanche index *k* and found optimal at intermediate 3–6 s window size values. Additionally, there are technical implications of using varying window sizes. When shorter time windows are considered, bandpass filter-induced correlations bias the estimation of temporal autocorrelations. In contrast, longer time windows lead to insufficient data points for the estimation of temporal correlations and diminished statistical power, especially for signals under 2 min. To test the impact of *f*E/I and DFA parameter combinations on the results, we devised models using a modified *f*E/I and DFA parametrization (Table S1).

#### Aperiodic exponent

Neural power spectra comprise of oscillatory, as well as aperiodic, 1/*f*-like components (Freeman and Zhai, 2009; He et al., 2010) oscillatory peaks rising above the aperiodic component (Donoghue et al., 2020). The 1/*f* exponent quantifies the scale-free, nonoscillatory background activity in the brain's power spectrum, independent of oscillatory peaks. Simulations have related this aperiodic exponent with E/I ratio, smaller exponents being putatively associated with a higher E/I ratio (Gao et al., 2017). Power decays with frequency with a 1/*f*^β^ function, with β the aperiodic exponent, determining the steepness of the power spectrum slope. We used spectral parameterization (*specparam*, former FOOOF) to compute the aperiodic exponent (Donoghue et al., 2020). This aperiodic exponent fitting range choice was motivated by the CROS (Critical Oscillations) model simulations (Poil et al., 2012; Avramiea et al., 2020; Avramiea et al., 2022). The simulations showed that networks operating near an excitation–inhibition (E/I) balanced regime may exhibit 1/*f*-like spectral scaling predominantly at frequencies below the dominant oscillatory peak typically located in the alpha range. When testing the ranges in which the 1/*f* slopes most closely reflect critical dynamics, the data suggested that spectral scaling in the low-frequency ranges (1–5 Hz) best captured a regime where E/I is balanced. In addition, as a technical consideration, we fit the exponent below the alpha peak to minimize conflation with oscillatory activity from alpha and beta peaks. Power spectra were fit between 1 and 5 Hz using the settings: peak_width_limits = [2, 12], max_n_peaks = 6, min_peak_height = 0.1.

#### Individual alpha frequency (IAF) fitting

To accommodate slower alpha activity in younger children, we used *specparam* to decompose the 1–40 Hz power spectral density (PSD) of each source and electrode time series into aperiodic, 1/*f* exponent, and periodic components, peak alpha frequency (PAF) and height above the aperiodic fit, quantifying the strength of oscillatory activity relative to the background 1/*f* activity. The PAF was defined as the center frequency of the largest detectable periodic peak between 6 and 14 Hz in the power spectrum. For this peak, *specparam* returns a modeled height *H*_log10_ representing the peak amplitude above the aperiodic background on a log-10 power scale_._ In the present study, this peak height was used as a measure of alpha peak strength, sometimes referred to as a signal-to-noise (SNR) index, larger values indicating a stronger oscillatory peak relative to the aperiodic component. For each channel or parcel, an individualized alpha band (IAF) was defined by symmetrically expanding ±2 Hz around the PAF to prevent excessively narrow or broad bands for DFA computation.

### Experimental design and statistical analysis

We computed Pearson’s *r* to assess correlations between age and EEG quality-related metrics (signal duration, peak height), age and IQ, and between sensorimotor and association *f*E/I and 1/*f* exponent values. Wilcoxon rank-sum tests compared sensorimotor–association *f*E/I and 1/*f* exponent values across age groups.

To examine associations between criticality-sensitive biomarkers and IQ across hierarchical regions, we first fitted simple linear regression models predicting sensorimotor and association *f*E/I and 1/*f* exponent from IQ, having collapsed hemispheres and alpha band frequencies (8.3–10.5 and 10.5–13.4 Hz) for simplicity. The models incorporated age and sex as covariates of no interest (i). Next, to study how individual variation in criticality-sensitive measures relates to IQ, we tested the associations between *f*E/I and 1/*f* exponent in the sensorimotor and association regions and IQ using multilevel models accounting for individual variability in *f*E/I (ii. a) and 1/*f* exponent (ii. b). Bilateral hemisphere observations (of the 1/*f* exponent) and additionally, readings pertaining to two alpha band frequencies (8.3–10.5 and 10.5–13.4 Hz) in case of *f*E/I, were considered for each subject, yielding 4 (ii b, iv a) and 8 observations (ii a, iii a) per subject for each of the two sensorimotor and association regions, respectively. Using multiple observations per subject elevated the statistical power of the multilevel (ii.) and GAMM (iii, iv) models. IQ-by-hierarchy interactions were further examined using Johnson–Neyman intervals to identify IQ ranges driving the effects. Multilevel model degrees of freedom were estimated using Satterthwaite approximation (R: lmerTest).

i. Y*_f_*_E/I-hierarchy, 1/*f* exp-hierarchy_ = β_0_ + β_1_ age_i_ + β_2_ sex_i_ + β_3_IQ_i_ + ε
ii. a) Y*f*E/I_ij_ = β_0_ + β_1_ age_i_ + β_2_ sex_i_ + β_3_ hemisphere_ij_ + β_4_ freq_ij_ + β_56_(hierarchy_ij_, IQ_i_) + (1|subject) + ɛ_ij_
b) Y1/*f* exp_ij_= β_0_ + β_1_ age_i_ + β_2_ sex_i_ + β_3_ hemisphere_ij_ + β_45_(hierarchy_ij_, IQ_i_) + (1|subject) + ɛ_ij​_

where Y*_f_*_E/I,_ Y_1/*f* exp_ are the *f*E/I, 1/*f* exp. values and β_1–4_ are the effects of age_i_, sex_i_, hemisphere_ij_, freq_ij_ covariates. β(hierarchy_ij_, IQ_i_) stands for the linear interactions between hierarchy_ij_ and IQ_i_ and their main effects. (1|subject) are the random subject intercepts, β_0_ is the intercept, and ɛ_ij_ is the error.

To answer whether age and cortical hierarchy modulate associations between the two criticality-sensitive biomarkers and IQ, we next examined age-dependent trajectories of these biomarkers across two broad hierarchical regions and seven functional networks in relation to IQ using generalized additive mixed models (GAMMs) with tensor product smoothing splines (Wood, 2017). The models captured interactions among age, large sensorimotor–association regions (models iii a, iv a), Yeo7 networks (models iii b, iv b), and IQ, in predicting *f*E/I and 1/*f* exponent, while accounting for repeated measures per subject. For the Yeo7 network models (iii b and iv b), bilateral hemisphere observations (for 1/*f* exponent) and additionally, observations pertaining to two alpha band frequency ranges in case of *f*E/I were considered for each subject across the seven Yeo7 networks. The dependent variables comprised network-by-hemisphere-by-subject values (1/*f* exponent: 7 × 2 × *N* = 128 subjects = 1,792 values) and network-by-hemisphere-by-frequency values (*f*E/I: 7 × 2 × 2 × *N* = 128 subjects = 3,584 values). In the hierarchy models (models iii a, iv a), *f*E/I and 1/*f* exponent values across the seven Yeo7 networks were averaged within sensorimotor and association regions (*f*E/I: 2 regions × 2 hemispheres × 2 frequencies × *N* = 128 subjects = 1,024 values; 1/*f* exponent: 2 regions × 2 hemispheres × *N* = 128 subjects = 512 values). Network rank (1–7) was treated as a continuous predictor reflecting sensorimotor-to-association ordering. All models included linear effects of sex, hemisphere, and additionally frequency range (*f*E/I) as covariates of no interest and subject as a random effect. Model complexity was assessed with the *gam.check* function, and smoothing penalties (option select = TRUE) enabled shrinkage of uninformative terms to zero. The GAMM models are summarized as follows (Ramjith et al., 2021; Ramjith et al., 2024):

iii. a) Y*f*E/I_ij_ = β₀ + β₁·sex_i_ + β₂·hemisphere_ij_ + β₃·freq_ij_ + β₄₅₆·(age_i_, hierarchy_ij_, IQ_i_) + f₄₅₆·(age_i_, hierarchy_ij_, IQ_i_) + s(subject_i_) + ɛ_ij_
b) Y*f*E/I_ij_ = β₀ + β₁·sex_i_ + β₂·hemisphere_ij_ + β₃·freq_ij_ + β₄₅₆·(age_i_, network_ij_, IQ_i_) + f₄₅₆·(age_i_, network_ij_, IQ_i_) + s(subject_i_) + ɛ_ij_
iv. Y1/*f* exp_ij_ = β₀ + β₁·sex_i_ + β₂·hemisphere_ij_ + β₃₄₅·(age_i_, hierarchy_ij_, IQ_i_) + f₃₄₅·(age_i_, hierarchy_ij_, IQ_i_) + s(subject_i_) + ɛ_ij_

Due to absence of age effects on the 1/*f* exponent, models were simplified by excluding age from interaction terms and retaining it as a linear main effect:

a) Y1/*f* exp_ij_ = β₀ + β₁·sex_i_ + β₂·hemisphere_ij_ + β₃·age_i_ + β₄₅·(hierarchy_ij_, IQ_i_) + f₄₅·(hierarchy_ij_, IQ_i_) + s(subject_i_) + ɛ_ij_
b) Y1/*f* exp_ij_ = β₀ + β₁·sex_i_ + β₂·hemisphere_ij_ + β₃·age_i_ + β₄₅·(network_ij_, IQ_i_) + f₄₅·(network_ij_, IQ_i_) + s(subject_i_) + ɛ_ij_

Where Y*f*E/I_ij_ , Y1/*f* exp_ij_ are the *f*E/I, 1/*f* exp. values, *f*(age_i_, hierarchy_ij_, IQ_i_) and f(age_i_, network_ij,_ IQ_i_) are the tensor product smooth interaction combinations of age_i_, hierarchy_ij_, network_ij_, IQ_i_. β·(age_i_, hierarchy_ij_, IQ_i_) stands for the linear interactions between age_i_, hierarchy_ij_, and IQ_i_ and their main effects. β_1–3_  are the effects of age_i_, sex_i_, hemisphere_ij_, freq_ij_ covariates. s(subject_i_) are the random subject intercepts, and β_0_ is the intercept and ɛ_ij_ is the error. Bonferroni’s correction was applied to account for the number of fixed and smooth effects tested per biomarker model. To explore fringe-IQ associations with criticality-sensitive biomarkers, we performed post hoc unpaired *t* tests comparing high-IQ (sample mean + SD) and low-IQ (sample mean − SD) subgroups. At the source level, 84% of DFA values exceeded 0.6 for *f*E/I computation after region averaging, prior to imputation, and 92% exceeded the 0.55 threshold. No missing 1/*f* exponent data were present (Table S1). Missing *f*E/I values were imputed using fast-chained random forests via the *R* package *missRanger* v2.6.1 (Stekhoven and Bühlmann, 2011). To control for potential imputation bias, all control analyses targeting sensor regions, *f*E/I computed on the individual alpha frequencies_,_ and alternative *f*E/I and DFA parametrizations were conducted without imputation (Table S1).

Analyses were conducted in RStudio v4.2.3 with R version 4.0 (Team, 2024).

## Results

### Near-critical dynamics in the association as opposed to sensorimotor regions are more strongly linked to a higher IQ

We enrolled 128 typically developing children (72 female), 6–19 years old, who completed resting-state EEG and cognitive testing (Fig. 1*A–D*). The sample had a full-scale IQ (Wechsler Intelligence Scale) of 111 ± 13 (range, 71–145). To test broad hierarchical differences, we collapsed the seven Yeo7 networks into association versus sensorimotor regions based on their position along the sensorimotor–association cortical hierarchy (Zhang et al., 2024; Fig. 1*C*). To index putative near-critical dynamics of ongoing oscillations, we derived the aperiodic 1/*f* exponent and *f*E/I criticality-sensitive biomarkers from resting EEG (Fig. 1*A*,*B*; Fig. S1*A*). Signal quality-related metrics showed no detectable association with age (number of clean segments: *r* = 0.02, *p* = 0.82; association-peak height: *r* = 0.16, *p* = 0.07; sensorimotor-peak height: *r* = 0.10, *p* = 0.25), indicating broadly comparable signal characteristics across the age range (Fig. S1*B–D*). Across regions, alpha *f*E/I and 1/*f* exponent were modestly, but significantly negatively correlated (association: *r* = −0.26, *p* = 0.004; sensorimotor: *r* = −0.19, *p* = 0.034; Fig. 1*E*,*F*).

We next characterized the observed operating ranges of both measures during eyes-open rest, including proximity of *f*E/I to the theoretical critical value of 1. Children exhibited an aperiodic 1/*f* exponent of 1.46 ± 0.34 (range 0.36–2.77) and a subcritical alpha *f*E/I of 0.87 ± 0.19 (range, 0.25–1.58), with regional variability in biomarker topographies (Fig. S1*A*).

**Figure 2.**
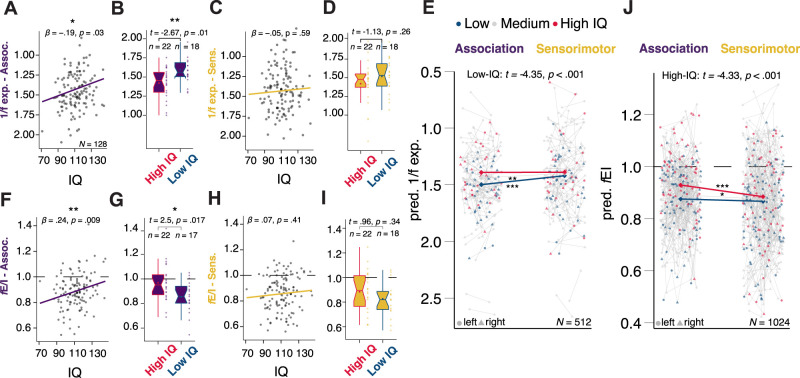
*f*E/I and 1/*f* exponent correlate with IQ in association but not sensorimotor regions, high-IQ children showing a more critical *f*E/I and a lower 1/*f* exponent. IQ exhibited significant correlations with the 1/*f* exponent and *f*E/I in aggregated association regions (***A***, ***F***), while the correlations in the aggregated sensorimotor regions were nonsignificant (***C***, ***H***). In the association cortex, high-IQ children (above sample mean + SD) had a lower 1/*f* exponent and an *f*E/I ratio closer to the theoretical critical value of 1 compared with low-IQ children (below sample mean − SD; ***B***, ***G***), whereas this distinction was not significant in sensorimotor regions (***D***, ***I***). ***E***, ***J***, Below- and average-IQ (sample mean − SD, IQ = 98) individuals had higher association in contrast to sensorimotor 1/*f* exponent values. Above- and average-IQ (sample mean + SD, IQ = 124) individuals had a higher association in contrast to sensorimotor fE/I ratio. Notes: Results depicted in ***A***, ***C***, ***F***, ***H*** pertain to linear regression models including age and sex as linear covariates (*N*_fE/I, 1/*f* exp._ = 128 subjects). Results in ***E***, ***J*** pertain to linear multilevel models including age, sex, hemisphere, and frequency range as covariates. Connecting gray lines in ***E***, ***J*** indicate measurements from the same subject: each of the *N* = 128 subjects had bilateral measurements (1/*f* exponent), and bilateral measurements in the 8.3–10.5 and 10.5–13.4 Hz frequency ranges (fE/I) for each sensorimotor–association region, leading to 4 and 8 measurements per subject, respectively, and *N*_1/*f* exp._ = 512 and *N*_fE/I_ = 1,024 measurements. Left–right hemisphere measurements are depicted as circles and triangles, respectively.

We examined whether markers of near-critical dynamics relate to IQ across association and sensorimotor cortical regions. In linear models controlling for age and sex, the aperiodic 1/*f* exponent in association cortex was significantly associated with IQ (*t*_(124)_ = −2.18, *β* = −0.19, *p* = 0.03, linear regression; Fig. 2*A*), whereas the corresponding association in sensorimotor regions was not significant (*t*_(124)_ = −0.54, *β* = −0.05, *p* = 0.59, linear regression; Fig. 2*C*). A multilevel model controlling for age, sex, and hemisphere effects revealed that the effect of IQ on the 1/*f* exponent depended on individual association and sensorimotor 1/*f* exponent values. In particular, a lower association 1/*f* exponent was associated with a higher IQ (*t*_(381)_ = 3.02, *p*_bonf_ = 0.009, multilevel model; also GAMM: *t*_(390)_ = 2.880, *p*_bonf_ = 0.029). This effect was prominent for below- and average-IQ children (IQ = 97, *t*_(384)_ = −4.35, *p* < 0.01; IQ = 111, *t*_(384)_ = 3.14, *p* < 0.01; Fig. 2*E*, Table S1). A subgroup of high-IQ children (1SD above sample average and higher, *n*_high_ = 22) had a lower 1/*f* exponent in the association cortex (1.41, SD 0.21), in contrast to low-IQ children (1SD below sample average and lower, *n*_low_ = 18; *t*_(38)_ = −2.67, *p* = 0.01, post hoc unpaired *t* test; Fig. 2*B*). This difference was nonsignificant in the sensorimotor cortices (*t*_(38)_ = −1.13, *p* = 0.26, post hoc unpaired *t* test; Fig. 2*D*).

A higher *f*E/I in the broad association regions, proximal to 1 in our largely subcritical sample (*f*E/I < 1), was significantly associated to a higher IQ, after controlling for age, sex effects in a linear model (*t*_(124)_ = 2.68, *β* = 0.24, *p* = 0.009, linear regression; Fig. 2*F*). Conversely, the association in the sensorimotor regions was not significant (*t*_(124)_ = 0.8, *β* = 0.07, *p* = 0.41, linear regression; Fig. 2*H*). A multilevel model controlling for age, sex, hemisphere, and low vs high alpha band effects showed that the association between *f*E/I ratio and IQ at low-, intermediate-, and low-IQ values depended on individual association and sensorimotor *f*E/I ratio values (*t*_(892)_ = −2.04, *p* = 0.04, multilevel model). The effect was most visible for above average-IQ children (IQ = 124: *t*_(892)_ = −4.33, *p* < 0.001; Fig. 2*J*, Table S1). A subgroup of high-IQ children (*f*E/I of 0.98*,* SD 0.17 and at least 1 SD above the sample average, *n*_high_ = 22) had an *f*E/I ratio closer to the theoretical critical value of 1 in the association cortex, compared with the low-IQ (1SD below the sample average and lower, *n*_low_ = 17) group (*t*_(37)_ = 2.5, *p* = 0.017, post hoc unpaired *t* test; Fig. 2*G*). On the other hand, the sensorimotor *f*E/I ratio did not significantly differ between high- and low-IQ children (*n*_low_ = 18, *t*_(38)_ = 0.96, *p* = 0.34, post hoc unpaired *t* test; Fig. 2*I*).

### The rank in the sensorimotor–association hierarchy moderated the association between 1/*f* exponent and IQ

**Figure 3.**
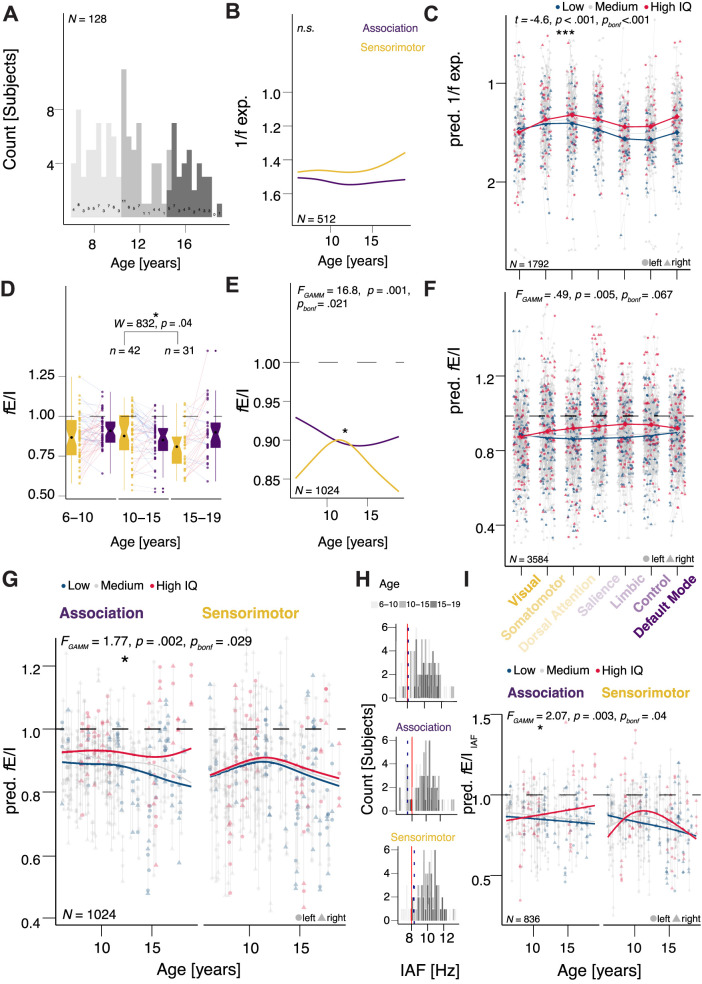
The sensorimotor–association rank moderated the association between 1/*f* exponent and IQ, while development and rank moderated the association between *f*E/I and IQ. ***A***, Age was uniformly distributed across subjects. ***B***, No age effects on 1/*f* exponent across the cortical hierarchy. ***C***, Cortical hierarchy rank moderated the 1/*f* exponent–IQ association, as indicated by a significant interaction between hierarchy and IQ. ***D***, ***E***, Sensorimotor *f*E/I peaked in the 10–15 years group, declining in the 15–19 group. ***E***, Nonlinear *f*E/I developmental effects in sensorimotor regions. ***F***, ***G***, The relationship between IQ and *f*E/I depends on age, and this dependence is specific to association cortex, as shown by a significant age × IQ interaction in the association cortex. ***H***, At the whole head level (top), 9% of children (dotted blue line) had an individual peak alpha frequency below 8 Hz (red line; association level: 17%; sensorimotor level: 6%). Red lines in ***H*** represent the 8 Hz threshold of the alpha-peak distribution in the 6–14 Hz interval at whole head, association, and sensorimotor region levels. The dotted dark blue line represents the 10% distribution threshold. ***I***, Age and cortical rank moderation of *f*E/I_IAF_–IQ associations is robust when individual alpha frequencies (IAF) are considered. Notes: *N*_fE/I, 1/*f* exp._ = 128 subjects. GAMMs were fitted at two spatial scales: seven Yeo7 networks (“network” models) and broad sensorimotor versus association regions (“hierarchy” models, with network values averaged within regions). Panels ***C***, ***F***, ***G***, and ***I*** show predicted 1/*f* exponent and fE/I values across Yeo7 networks (***C***, ***F***) or across age by hierarchy (***G***, ***I***) at three IQ levels: mean (“Medium IQ”, gray), mean + SD (“High IQ”, red), and mean − SD (“Low IQ”, blue). Dependent variable structure. 1/*f* exponent: network × hemisphere × subject = 7 × 2 × 128 = 1,792 values. fE/I: network × hemisphere × frequency × subject = 7 × 2 × 2 × 128 = up to 3,584 values, depending on missingness. Hierarchy fE/I: region × hemisphere × frequency × subject = 2 × 2 × 2 × 128 = up to 1,024 values, depending on missingness. Models controlled for hemisphere, sex, and frequency band (*f*E/I and *f*E/I_IAF_). *f*E/I_IAF_ models additionally controlled for peak height and were fitted on nonimputed data. *F* statistic and *p* values from R:mgcv for penalized regression splines are reported for the smooth GAMM terms. Bonferroni-corrected *α* = 0.007 (1/*f*), *α* = 0.003 (*f*E/I).

We showed that near-critical brain dynamics in broad association regions may be more strongly linked to a higher IQ. As these dynamics and cognitive functions are known to evolve with age, we investigated the way in which the age-dependent variation in *f*E/I and 1/*f* exponent across the two broad hierarchical association–sensorimotor regions and the seven networks, relates to IQ. Age was uniformly distributed across subjects (Fig. 3*A*). We record a significant nonlinear age effect on *f*E/I in the sensorimotor (*F*_GAMM(3)_ = 16.88, *p*_bonf_ = 0.021, GAMM), but not the association regions (*F*_GAMM(3)_ < 0.01, *p* = 0.56 GAMM; Fig. 3*D*,*E*; Table 1), unlike for the 1/*f* exponent, where we found no age effects in either region (association: *F*_GAMM(3)_ < 0.01, *p* = 0.45; sensorimotor: *F*_GAMM(3)_ = 4.51, *p* = 0.19, GAMM; Fig. 3*B*). The effects of IQ at high-, average-, and low-IQ values on the 1/*f* exponent in the seven Yeo7 networks at high-, average-, and low-IQ values, after controlling for age, sex, and hemisphere effects, are shown in Figure 3*C*. The Yeo 7 network rank significantly moderated the association between 1/*f* exponent and IQ, the association between the 1/*f* exponent and IQ varying systematically along the sensorimotor–association axis, model predictions suggesting a stronger IQ-related separation of lower versus higher exponent values from sensorimotor to association networks (*t*_(1661)_ = −4.6, *p*_bonf_ < 0.001, GAMM; Figs. 2*E*, 3*C*; Table S1). The hierarchy moderation effects were validated when the analysis was conducted in sensor space on broadly analogous sensorimotor–association regions [region × IQ: *t* = −4.02, *p*_bonf_ = 0.001; (local) region × IQ: *t* = −3.26, *p*_bonf_ = 0.008, GAMM; Table S1], and when using a Desikan–Killiany parcellation (*t* = −7.18, *p*_bonf_ < 0.001, GAMM), suggesting the effects do not depend on the source reconstruction pipeline or parcellation (Table S1).

**Table 1.** Significant parametric and smooth terms of 1/*f* exponent and *f*E/I GAMM models

Model			Effect	Statistic		*p*_bonf_
1	1/*f* exponent	Hierarchy	IQ × hierarchy	*t*	2.880	0.029
2		Network	IQ × network (1–7)		−4.6	<0.001
3	*f*E/I	Hierarchy	Age × hierarchy (sensorimotor)	*F*_GAMM_	16.880	0.021
			Age × IQ × hierarchy (association)		1.770	0.029
4		Network	Network (1–7)		2.830	0.03
			Age × network (1–7)		0.660	0.015
			Age × IQ × network (1–7)		0.290	0.017
5	*f*E/I_IAF_	Hierarchy	Age × hierarchy (sensorimotor)		17.263	0.009
			Age × IQ × hierarchy (association)		2.073	0.04
6		Network	Age × IQ × network (1–7)		0.612	<0.001

1−2, 1/*f* exponent. The sensorimotor–association axis rank moderated the association between 1/*f* exponent and IQ in both broad region (1) and Yeo7 network (2) models. 3–6, *f*E/I_(IAF)_. 3, 5, The relationship between IQ and *f*E/I_(IAF)_ depends on age and cortical hierarchy rank, and this dependence is specific to the association cortex, as shown by a significant age × IQ interaction in the association cortex. 4, 6, When considering the seven Yeo7 networks, age, and network rank moderated the association between *f*E/I_(IAF)_ and IQ, model predictions suggesting a stronger IQ-related delineation of low versus high *f*E/I_(IAF)_ values during adolescence, particularly in association cortex networks. Notes: *N* = 128 subjects. GAMMs were fitted at two spatial scales: broad sensorimotor vs association regions (“hierarchy” models) and seven Yeo7 networks (“network” models). “Rank” denotes the position of the Yeo7 networks along the sensorimotor–association axis. The 1/*f* exponent dependent variable consisted of network-by-hemisphere-by-subject values (7 × 2 × *N* = 128 subjects = 1,792 values). In the hierarchy model, these values were averaged within sensorimotor and association regions (2 regions × 2 hemispheres × *N* = 128 subjects = 512 values). The *f*E/I dependent variable consisted of network-by-hemisphere-by-frequency subject values (7 × 2 × 2 × *N* = 128 subjects = up to 3,584 values, depending on missingness). In the hierarchy model, these values were averaged within sensorimotor and association regions (2 regions × 2 hemispheres × 2 frequencies × *N* = 128 subjects = up to 1,024 values, depending on missingness). Models controlled for hemisphere, sex, and frequency band (*f*E/I and *f*E/I_IAF_). *f*E/I_IAF_ models additionally controlled for peak height and were fitted on nonimputed data. *F* statistic and *p* values from R:mgcv for penalized regression splines are reported for the smooth GAMM terms. Bonferroni-corrected *α* = 0.007 (1/*f*), *α* = 0.003 (*f*E/I).

### Age and rank in the sensorimotor–association hierarchy moderated the association between *f*E/I and IQ

As shown in the previous section, the cortical hierarchy effect on sensorimotor *f*E/I nonlinearly depended on age (*F*_GAMM(3)_ = 16.88, *p*_bonf_ *=* 0.021, GAMM; Table 1). *f*E/I measures in *sensorimotor* regions lower in the cortical hierarchy, for example, visual, somatomotor, and dorsal attention, peaked (were highest, on average) in the mid-childhood, 10–15 years group, declining in the adolescent, 15–19 years group (*W* = 832, *p* = 0.04, Wilcoxon rank-sum test; Fig. 3*D*,*E*).

We next asked if age-dependent changes in *f*E/I moderate the association between cortical hierarchy and IQ. The effects of IQ at high-, average-, and low-IQ values on *f*E/I in the two broad hierarchical regions and seven Yeo7 networks are depicted in Figure 3*F*,*G* and Figure S2*A*, respectively. Age significantly moderated the association between *f*E/I and IQ in association networks (*F*_GAMM(63)_ = 1.77, *p*_bonf_ = 0.029, GAMM; Fig. 3*G*, Table 1, Table S1), model predictions suggesting a stronger IQ-related delineation of low versus high *f*E/I values during adolescence in association regions. When considering the Yeo 7 networks, age and network rank significantly moderated the association between *f*E/I and IQ (*F*_GAMM(63)_ = 0.29, *p*_bonf_ = 0.017, GAMM), model predictions suggesting a stronger IQ-related delineation of low versus high *f*E/I values during adolescence, particularly in association cortex networks (salience, limbic, control; Table 1, Fig. S2*A*, Table S1). The age-by-hierarchy moderation effects were validated in sensor space, without imputation, and while controlling for peak height [age × region × IQ: *F*_GAMM(15)_ = 13.77, *p*_bonf_ = 0.006; age × (local) region × IQ: *F*_GAMM(63)_ = 0.57, *p*_bonf_ = 0.001, GAMM], suggesting the effects do not depend on the source reconstruction pipeline (Table S1).

### *f*E/I–IQ associations are robust when individual alpha frequencies (IAF) are considered

To accommodate slower alpha activity in younger children, we recomputed *f*E/I using each subject’s individual alpha frequency (*f*E/I_IAF_). A minority of children (9%) showed a whole-head average peak alpha frequency below 8 Hz (Fig. 3*H*). All IAF-based models controlled for alpha peak height and were fitted on nonimputed datasets. The cortical hierarchy effect on *f*E/I_IAF_ nonlinearly varied with age (region-level: *F*_GAMM(3)_ = 17.263, *p*_bonf_ *=* 0.009; network-level: *F*_GAMM(15)_ = 0.88, *p*_bonf_ *=* 0.08, GAMM; Table 1, Table S1). The effects of IQ at high-, average-, and low-IQ values on *f*E/I_IAF_ in the two broad hierarchical regions and seven Yeo7 networks are depicted in Figure 3*I* and Figure S2*B*, respectively. Age significantly moderated the association between *f*E/I_IAF_ and IQ in association networks (*F*_GAMM(16)_ = 2.07, *p*_bonf_ = 0.04, GAMM), model predictions suggesting a stronger IQ-related delineation of low versus high *f*E/I_IAF_ values during adolescence in association regions (Fig. 3*I*, Table 1, Table S1). When considering the seven Yeo 7 networks, age and network rank significantly moderated the association between *f*E/I_IAF_ and IQ (*F*_GAMM(63)_ = 0.61, *p*_bonf_ <0.001, GAMM), model predictions suggesting a stronger IQ-related delineation of low versus high *f*E/I_IAF_ values during adolescence, particularly in association cortex networks (salience, limbic, control; Fig. S2*B*). Furthermore, the age-by-hierarchy moderation effect was preserved under a Desikan–Killiany parcellation (*F*_GAMM(63)_ = 0.59, *p*_bonf_ = 0.003, GAMM; Table S1), suggesting the effects do not depend on parcellation. To test the impact of *f*E/I and DFA parameter combinations on the results, we devised *f*E/I_IAF-modparam_ models under a modified DFA and *f*E/I parametrization and no imputation. Across all tested window sizes (3–6 s) and overlaps (75–85%), the age-by-hierarchy moderation effect was preserved under the control models (7C: *F*_GAMM(15)_ = 6, *p*_bonf_ < 0.001; 8C: *F*_GAMM(15)_ = 6.57, *p*_bonf_ < 0.001; 9C: *F*_GAMM(15)_ = 2.77, *p*_bonf_ = 0.04; 10C: *F*_GAMM(15)_ = 2.41, *p*_bonf_ = 0.02, GAMM; Table S1).

## Discussion

### Association cortex near-critical dynamics are associated with intelligence in children

We investigated the relationship between intelligence and two EEG proxies of critical brain dynamics: functional excitation/inhibition ratio (*f*E/I) and the 1/*f* aperiodic exponent across the seven Yeo7 cortical networks (Yeo et al., 2011). The regions were previously functionally ranked from lower-order sensorimotor, to higher-order, association networks (Sydnor et al., 2021; Sydnor et al., 2023; Zhang et al., 2024). We found near-critical dynamics, as quantified using *f*E/I ratio and 1/*f* exponent biomarkers, to be more strongly associated with a higher IQ in association than in sensorimotor regions. The high-IQ subgroup had a mean association *f*E/I close to the theoretical critical threshold of 1 (0.98), in line with the notion that a balanced association network E/I ratio characterizes a high intelligence. Multilevel models indicated that, while the 1/*f* exponent was most predictive of below-average intelligence, *f*E/I was most predictive of above-average intelligence, suggesting a complementary role of the two criticality-sensitive biomarkers in predicting intelligence. Importantly, the moderation of *f*E/I IQ associations by age and cortical hierarchy rank was robust across multiple control models, supporting a specific contribution of *f*E/I in intelligence.

Our findings regarding associations between *f*E/I and 1/*f* exponent and intelligence align with fMRI-based edge-of-chaos and avalanche criticality measures previously being associated with greater fluid intelligence criticality (Ezaki et al., 2020; Xu et al., 2022), albeit our findings were most prominent in association regions. Computational modeling studies previously suggested that critical neural networks striking a certain balance between excitation and inhibition tend to exhibit intermediate *f*E/I values, which are thought to maximize dynamic range and information propagation (Bruining et al., 2020; Avramiea et al., 2022). The *f*E/I metric approaches 1 at the excitation–inhibition balance point, marking the critical regime and was shown to maximize LRTC (DFA), a proxy of criticality. Empirically, a lower *f*E/I in contrast to healthy controls was observed in genetic neurodevelopmental disorders, which are frequently marked by intellectual disability. Ramautar et al. (2025) recorded a low alpha *f*E/I in children with a GRIN2B disorder. Houtman et al. (2021) similarly found a low *f*E/I in contrast to healthy controls, albeit in the beta frequency in STXBP1 disorder children.

We found a lower association-cortex 1/*f* exponent to be associated with a higher IQ. To the best of knowledge, there is no published consensus on a “critical” value of the 1/*f* exponent. Preliminary simulations suggested that, for the fitting range used in this study, intermediate ranges (1.25–1.5) may correspond to a balanced E/I, whereas exceedingly high or low exponents may correspond to sub- and super-critical regimes, respectively, consistent with prior findings (Gao et al., 2017). Although methodological heterogeneity precludes close comparisons, our study broadly echoes previous findings of a flatter (lower) exponent being associated with enhanced cognitive performance. Ostlund et al. (2021) found faster reaction times and drift rates, implying evidence being gathered more rapidly and clearly, leading to quicker and more accurate decisions, in subjects with a lower 1/*f* exponent. Furthermore, flatter aperiodic slopes were associated with increased recognition during task acclimatization (Immink et al., 2021).

Crucially, while we interpret *f*E/I close to 1 as “more consistent with critical dynamics,” and a lower 1/*f* exponent as “more consistent with super-critical dynamics, or a higher E/I”, we recognize that the methodological challenges in assessing proximity to criticality and study heterogeneity preclude strong interpretations of either biomarker. As such, our conclusions center around relative differences across regions, and developmental stages, rather than absolute thresholds.

Additionally, our findings support a moderating effect of the rank on the association–sensorimotor axis (Sydnor et al., 2021; Sydnor et al., 2023) on the association between 1/*f* exponent and intelligence, whereby the associations between the 1/*f* exponent and IQ varied systematically along a hierarchical axis from the sensorimotor to the association cortex. This finding echoes previous studies that have suggested an enhanced role of the association regions in cognitive performance (Zhang et al., 2024), supporting the consideration of EEG criticality markers across ordered hierarchical networks. Furthermore, our finding regarding the strongest correlations being in the association regions aligns with the parietofrontal integration theory of intelligence, purporting that integration of frontal regions, including dorsolateral prefrontal cortex and anterior cingulate regions, plays an important role in intelligence (Jung and Haier, 2007; Vakhtin et al., 2014).

### Interplay of developmental effects on critical across cortical hierarchical regions in intelligence

The age-dependent *f*E/I changes observed in the sensorimotor cortex were nonlinear, in contrast to the association cortex in our sample, with sensorimotor networks showing a peak at intermediate age groups (10–15 years). The nonlinear sensorimotor developmental findings reflect previous findings recording nonlinear developmental effects across cortical regions, where visual and somatomotor cortices are thought to develop first, followed by association networks (Sydnor et al., 2021; Larsen et al., 2022; Sydnor et al., 2023; Zhang et al., 2024). Such nonlinear changes may reflect anatomical and temporal variation in cortical specialization and maturation. The developmental changes in the 1/*f* exponent were not significant; we observed only a decreasing trend toward a flatter (lower) exponent restricted to sensorimotor regions. This trend may reflect a faster development of the 1/*f* exponent in the sensorimotor networks, several studies recording a developmental flattening of the 1/*f* exponent (Voytek et al., 2015; Cross et al., 2025).

Age and rank on the association–sensorimotor axis moderated the association between *f*E/I and IQ, suggesting a nonlinear interplay of developmental *f*E/I effects across cortical hierarchical regions in intelligence. Modeling *f*E/I trajectories revealed a stronger IQ-related delineation of low versus high *f*E/I during adolescence, particularly in association cortex networks. This suggests that EEG criticality proxy biomarkers in association networks may be linked to individual differences in intelligence in an age-dependent manner, consistent with the hypothesis that the developmental modulation of critical dynamics across the cortical hierarchy may support more efficient cognitive processing.

### Limitations

The present study employed a cross-sectional design that enabled the investigation of criticality biomarkers across a broad childhood age range. While this design did not allow the characterization of within-individual developmental trajectories, this was not the primary aim of the study. Rather, our objective was to examine associations between intelligence and criticality markers in a neurotypical pediatric population taking into account sources of variability in critical brain dynamics in childhood. Future longitudinal studies may provide further insights into individual developmental trajectories of critical dynamics and their link to intelligence. Importantly, an intrinsic limitation given the observational design of the study is that the observed associations between the criticality-sensitive measures and intelligence may not be interpreted as causal evidence.

Furthermore, rather than a pure information processing proxy, the short IQ form used in this study spans domains of cognitive ability related to both fluid and verbal intelligence. As such, our results are to be interpreted as associations between critical brain dynamics and general cognitive performance. Performance may additionally be influenced by nonspecific factors such as testing familiarity, motivation, or socioeconomic status. Future explorations of IQ test dimensions with specific information processing demands will facilitate more direct inferences on the relationship between critical brain dynamics and information processing performance.

In addition, the analysis focused on resting-state EEG. The resting state is particularly relevant for self-organized criticality and intrinsic brain dynamics from which task-related computations emerge. Consistent with this, prior work shows that resting M/EEG critical dynamics covary with behavioral scaling laws and predict interindividual variability in auditory and visual detection (Palva et al., 2013) and motor performance (Smit et al., 2013). Additionally analyzing active paradigms and nonresting frequency bands may reveal further links between criticality modulation and task performance.

The presence of individual MRI recordings, or a pediatric head template, would have enhanced the spatial precision of the source localization presently conducted using an adult template. Importantly, the sensor-space control analyses reproduced the present source-reconstruction results, suggesting that the effects are detectable without source modeling, therefore unlikely to be an artifact of a particular source reconstruction pipeline. In addition, the analyses in this study intentionally focused on relatively large cortical regions (Yeo7 networks), reducing the probability that a millimeter-level optimized source model would markedly impact results.

Finally, while association regions such as the parietal cortex emit oscillations, other association regions may not show prominent oscillations. In such cases, the alpha bandpass used in *f*E/I computation may capture activity not uniquely attributable to oscillations, while interpretations of *f*E/I as an E/I-sensitive metric are strongest under a detectable oscillation scenario. To solidify interpretation, we recomputed *f*E/I using individualized alpha bands centered on each parcel's peak alpha frequency (PAF ± 2 Hz), where peaks present, and additionally modeled alpha peak height as a covariate, verifying our previous results, which suggests that the stronger association region findings were not driven by nonoscillatory phenomena alone.

### Conclusion and outlook

Our results help inform developmental values of *f*E/I and aperiodic exponent and their associations to intelligence and may provide guidelines for assessing the developing brain. Neurodevelopmental disorders, where timely interventions are needed, may benefit from early assessments of brain criticality-sensitive markers as cognitive biomarkers. Furthermore, through its findings of cortical hierarchy moderation of the association between criticality-sensitive markers and IQ, our study supports the consideration of criticality-sensitive markers across ordered hierarchical networks and helps to further specify the hypothesis that maturing networks approach a critical dynamic regime optimizing cognitive function.
